# Spiritual Pain in Meals on Wheels’ Clients

**DOI:** 10.3390/healthcare3040917

**Published:** 2015-10-10

**Authors:** Lisa Boss, Sandy Branson, Stanley Cron, Duck-Hee Kang

**Affiliations:** School of Nursing, The University of Texas Health Science Center, Houston, TX 77030, USA; E-Mails: sandra.m.branson@uth.tmc.edu (S.B.); stanley.cron@uth.tmc.edu (S.C.); duck-hee.kang@uth.tmc.edu (D.H.K.)

**Keywords:** spiritual pain, aging, biobehavioral, stress, depression, loneliness, salivary biomarkers

## Abstract

*Background*: Meals on Wheels’ clients are at risk for spiritual pain due to advanced age, social isolation, and failing health. They are also prone to stress, depression, and loneliness, placing them at risk for adverse biological disruptions and health outcomes. The purpose of the study was to examine associations of spiritual pain with psychosocial factors (stress, depression, loneliness, religious coping) and salivary biomarkers of stress and inflammation (cortisol, IL-1β) in Meals on Wheels’ clients. *Methods*: Data were collected cross-sectionally from 88 elderly (mean age 75.4). Spiritual pain, stress, depression, loneliness, and religious coping were measured with standardized instruments, and salivary biomarkers were assessed with enzyme immunoassays. *Results*: Spiritual pain was significantly and positively correlated with stress (*r* = 0.35, *p* ≤ 0.001), depression (*r* = 0.27, *p* = 0.01), and negative religious coping (*r* = 0.27, *p* = 0.01). Correlations with loneliness, positive religious coping, and salivary biomarkers were non-significant. *Conclusion*: Spiritual pain is an important concept in this population. Research should be expanded to understand the significance of spiritual pain in conjunction with psychosocial and biological variables and its potential impact on physical, mental, and cognitive health outcomes in the elderly.

## 1. Introduction

More than 60% of elderly Americans report that religion is an important part of their lives [1]. Many Americans also believe that religion and/or spirituality has a positive influence on both mental and physical health [2]. Forty three percent of a national random sample were found to pray for health reasons and 25% asked others to pray for their health [3]. In addition, 43% of elderly regularly attend church services [4] and 68% participate in private religious activities [5]. The prominent use of religion and spirituality, coupled with an increased number of health problems in the elderly justifies further research for their associations with other psychological variables and potential biological mechanisms.

Although religion and spirituality are closely related and are often used interchangeably, they are conceptually different. Religion refers to beliefs, behaviors, rituals, and ceremonies that are related to God or another higher power, can be practiced in public or private settings, and comes from established traditions that were developed over time in a community setting [2]. Spirituality, on the other hand, is the search for meaning and direction in life. Meaning in life is beyond the physical world, and is related to a greater being that can be God, another religious deity, or the universe [6]. People may nurture their spiritual being or neglect to develop their spirituality at all, but most are aware of the spirituality and continue to develop it over the course of their lifetime [6]. In an extensive and comprehensive review of over 1200 empirical studies [2,7], more than 90% of the studies have documented significant and positive findings of religion and/or spirituality on health. Potential mechanisms for the robust connection between religion, spirituality, and health were hypothesized to be via changes in health-related behaviors, social support, positive emotions, healthy beliefs, and positive expectations [7]. In addition, the mind-body interactions including biological mechanisms may have significant roles but have not been adequately examined [7].

### 1.1. Spiritual Pain

When a person is faced with a crisis, such as a life threatening illness, he/she may begin to question, wonder, or struggle to find meaning in life [6], and it is not uncommon for a person to question spiritual beliefs [8]. Common questions or expressions are, “Why me?” “Why does God allow this suffering?” “This is punishment”, or “But I’ve led a good life”. When these questions go unanswered internal conflicts arise and create a disruption between spiritual beliefs and what is going on in real life, ultimately leading to feelings of spiritual pain [2]. Spiritual pain has been defined as “a disruption in the principle which pervades a person’s entire being and which integrates and transcends one’s biological nature” [9], as “a pain caused by extinction of the being and meaning of self” [10], or as “a pain deep in your soul (being) that is not physical” [11]. Despite slightly different definitions, scientists generally agree that spiritual pain is a state of disorder and occurs when a person experiences a conflict, either acute or chronic, between their spiritual beliefs and actual life events [6]. Risk factors for spiritual pain include irreconcilable loss, physical or mental diminishment, material loss, loss of a relationship, or impending loss of one’s life [6]. Deep feelings of loneliness, abandonment, hurt from loved ones, and social isolation are other risk factors. Symptoms of spiritual pain include disconnection or unwillingness to engage with others, preoccupation with self, expressing a loss of the future, and feeling outcast, alone, abandoned, trapped, distress, despair, anger, shame, or guilt [6].

Unlike religion and spirituality, few researchers have specifically and empirically examined spiritual pain as it relates to any aspect of health. Mako *et al*. (2006) measured spiritual pain in relation to physical pain, symptom severity, and emotional distress in end-stage cancer patients and reported that greater spiritual pain was significantly and positively correlated with depression, but not physical pain or severity of illness [12]. Delgado-Guay *et al*. (2011) also measured spiritual pain in advanced cancer patients in relation to symptom expression, coping strategies, and quality of life and reported significant and positive findings of spiritual pain and physical/emotional symptoms [11]. In a qualitative study, cancer patients made statements about spiritual pain and its relationship with feelings of anger, frustration, and sense of unjustness as it related to the diagnosis of a serious illness [13]. Some researchers have examined other similar negative concepts of spirituality, such as spiritual distress, spiritual problems, and religious struggle, as they relate to overall well-being and health. Research findings show that similar negative spiritual concepts are related to greater psychological distress in cancer patients [14], as well as healthy individuals [15,16]. In addition, researchers report that participants with greater negative spiritual perceptions have poor physical health outcomes and higher mortality rates [17,18]. The lack of studies specifically focused on spiritual pain and health is clearly lacking, particularly in populations other than cancer patients.

### 1.2. Stress, Depression, Loneliness, and Religious Coping

The elderly are particularly at risk for psychological stress, depression, and loneliness, and these psychosocial states are interrelated. Empirical evidence delineates the role of psychological stress in the development, expression, and exacerbation of depression [19]. Epidemiological research indicates that psychological stress may induce depressive symptoms and is associated with poorer health outcomes and increased morbidity, particularly in the elderly population [20]. The uncontrollability of perceived stressors is predominantly associated with physiological, cognitive, and motivational consequences including increased vulnerability to depression [21,22]. Loneliness is interrelated with chronic stress and depression and often occurs concurrently with a sense of losing independence in the elderly population [23]. When lonely, elders typically experience negative thoughts and cannot focus on positive expectations, resulting in reduced confidence in others, fear, anger, and tension. The persisting negative thoughts and emotions promote stress and depression, further worsening the sense of loneliness [24,25].

Religious coping refers to the use of religious or spiritual practices to cope with stress and other negative psychosocial feelings and may include prayer, meditation, pastoral support, benevolent religious reframing, and religious faith. Positive religious coping occurs when a person uses religious activities and/or spiritual beliefs to find meaning, gain control, gain comfort and closeness to God or another religious deity during difficult situations [26]. The final result of using positive religious coping strategies is a better ability to manage difficult situations and less feelings of stress and depression [2]. Negative religious coping occurs when a person believes they are being punished, deserted, not loved, or abandoned by God or another religious deity. The person may also believe that their difficult or stressful situation is the work of the Devil or another negative religious deity. In this case, the result of using negative religious coping strategies is less ability to manage the difficult situation and more feelings of stress and depression [26].

### 1.3. Biological Influence

Although many elderly cope well using various resources, including religious coping strategies, some experience a prolonged and unresolved negative emotional state eliciting biological disruptions, such as sustained hypercortisolism and increased inflammation. Chronic psychological stress, depression, and loneliness are associated with dysregulation of the hypothalamic pituitary adrenal (HPA) axis. The hypothalamus is activated by a perception of emotional distress, stimulating increased levels of cortisol from the adrenal cortex. Physiological dysregulations occur during times of chronic stress and depression, in which the body is unable to maintain efficient balance, triggering impaired negative feedback of the HPA axis [27]. Ultimately, “biological aging” and cellular damage is promoted during times of chronic stress, resulting in accelerated metabolic changes seen throughout the body [27]. For example, hypercortisolism is a biological link associated with stress and decreased cognitive function in the elderly [28,29]. Additionally, Interleukin-1β (IL-1β) is a proinflammatory biomarker, which is associated with adverse physical and cognitive health outcomes in adults with high stress [30,31]. Loneliness also is associated with poor emotional and physical well-being, including increased morbidity and mortality risk, functional limitations, and poor health practices [32,33,34], and higher cortisol levels in adults [35,36]. Despite an identified link between psychosocial variables and adverse health outcomes, potential biological mechanisms underlying the relationship need to be examined further.

The influence of spirituality on health is less well understood [37]. In a systematic review of 114 studies, a positive correlation between spirituality and well-being, hope, and optimism was reported in 80% of the studies [38]. In 65% of the studies, negative correlations of spirituality and depression were found, and in 51% of the studies negative correlations of spirituality and anxiety were found [38]. In few studies, the relationship of spiritualty was examined in relation to immune and endocrine parameters. Spiritualty was negatively correlated with cortisol in adults [39,40] and positively correlated with interleukin-6 [41]. Similarly, spirituality was positively correlated with CD-4 count in human immunodeficiency positive adults [42] and with the number of lymphocytes and natural killer cells [43,44]. Although these findings suggest the potential impact of spirituality on biological responses, more studies incorporating biological responses are in clear need.

### 1.4. Theoretical Framework

**Figure 1:**
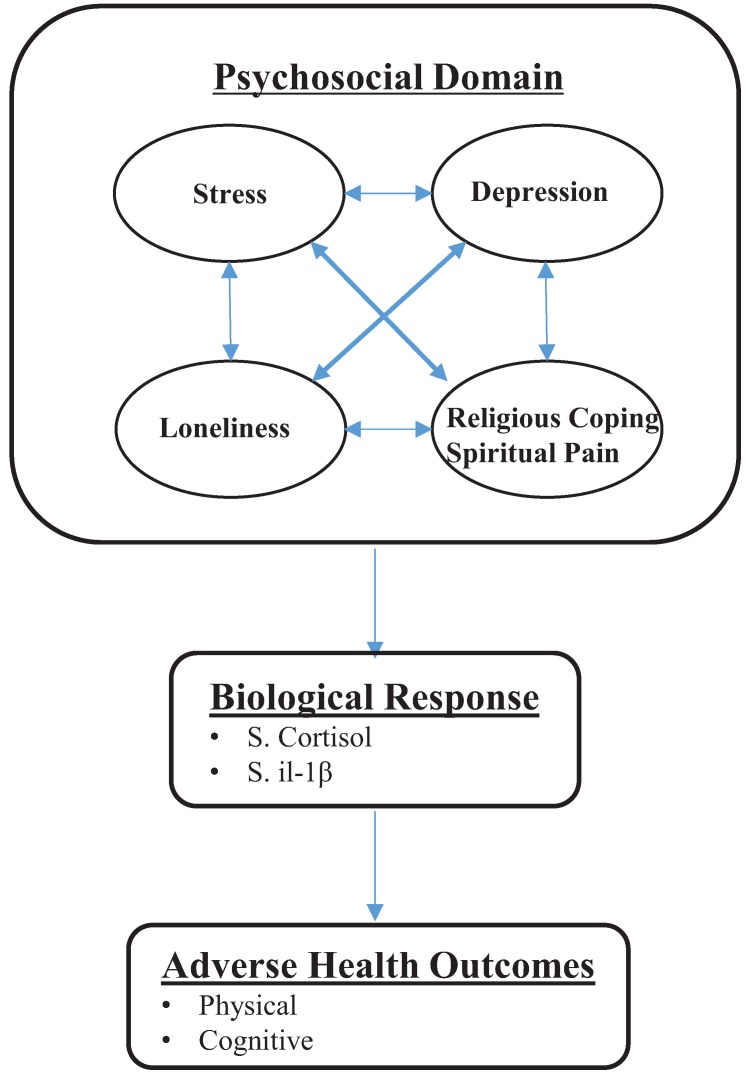
Adapted biobehavioral model for Meals on Wheels’ clients.

A biobehavioral interaction model [45] was used to guide this study, providing an overview of the relationships among psychosocial (stress, depression, loneliness, religious coping, and spiritual pain) and biological factors (cortisol and IL-1β), and health outcomes. In the overall model, health outcomes are posited to be influenced by a variety of factors, including individual, psychosocial, behavioral, and environmental factors, which can shape biological responses, and then ultimately affect mental, physical, and cognitive health outcomes [45]. A simplified conceptual model of this study is illustrated in Figure 1.

### 1.5. Purpose and Hypothesis

The purpose of this study was to examine spiritual pain from a biobehavioral perspective to understand associations with psychosocial factors (stress, depression, loneliness, and religious coping), and salivary biomarkers of stress and inflammation (cortisol and IL-1β) in Meals on Wheels’ (MOW) clients. It was hypothesized that spiritual pain would correlate positively with psychosocial factors, negative religious coping, salivary biomarkers, and correlate negatively with positive religious coping.

## 2. Experimental Section

### 2.1. Design and Sample

In a cross-sectional, descriptive study, 88 MOW clients (mean age 75.4 years) participated in the study in the fall of 2014. Sample size was based on power analysis with alpha set at 0.05, correlation between 0.3 and 0.4, and the statistical power of 0.8. Inclusion criteria were ≥60 years of age, ability to read/understand English, not diagnosed with a neurodegenerative disease, and currently enrolled in the MOW program in Montgomery County, TX, USA. Exclusion criteria were inability to complete psychometric instruments as instructed, inability to provide saliva sample, and currently taking hormone replacement therapy or corticosteroids.

Meals on Wheels’ clients are at risk for spiritual pain due to advanced age, social isolation, and presence of chronic illness. To qualify for the MOW program, seniors must be ≥60 years and without access to adequate nutrition, primarily due to physical and/or mobility limitations. Most MOW clients are primarily homebound and socially isolated from the community [46]. MOW clients are an ideal population for this study because they are ethnically diverse, live below the poverty line, and perceive their health to be fair or poor [47]. Further, community dwelling elderly who are socially isolated are at high risk for stress, depression, and loneliness [48,49].

### 2.2. Setting and Recruitment

The setting for the study was in a rural county in Texas where a charitable organization provides MOW services to disadvantaged elderly persons on a daily basis. Recruitment flyers were hand delivered by MOW volunteers to all approximately 500 MOW clients during their routine meal delivery over 8–10 weeks. Flyers included simple essential information about the study and the researcher’s contact information. When contacted, the researcher explained the study more in detail, answered all questions, and made an appointment to meet the client in their home to obtain informed consent and data collection. The study was approved by the Committee for the Protection of Human Subjects at the University. Each participant received a $ 10 gift card to WalMart upon completion of the study.

### 2.3. Measurement

Data were collected using standardized questionnaires (psychosocial and biological data) and saliva (biological data) in the participant’s home. Standard demographic information was collected in a self-report manner and included age, gender, race, height, weight, marital status, education, medical history, and medication list. Data collection took approximately 30–45 min and was performed by individuals specifically trained for data collection in this study. Participants were instructed to complete psychometric instruments prior to saliva collection. If the participant was unable to independently complete psychometric instruments the questions were read out loud by the data collector and the participant verbally reported the answers, which were then transcribed onto the instruments.

Spiritual pain was operationally defined for all participants as, “a pain deep in your soul (being) that is not physical.” It was then measured by asking, “Do you think you are experiencing spiritual pain now and how would you rate your overall spiritual pain?” Participants were asked to rate the pain on a 4-point Likert style scale from 1 (none) to 4 (worst). The measurement of spiritual pain in this manner is not formally validated, but ranking on a Likert-style scale is the standard form of measurement reported in previous studies [11,12].

Stress was measured with the Perceived Stress Scale (PSS), a 10-item questionnaire designed to assess the degree to which situations in a person’s life are appraised as stressful [50]. The Likert style, 10-item instrument consists of easy to understand questions and has been psychometrically tested in various populations. The item scores are summed and a higher score indicates a higher perceived stress level with the possible score range of 0–40. The PSS is considered a reliable instrument in elderly adults with a Cronbach’s α of 0.76 [51]. Cronbach’s α was 0.83 in this study.

Depression was measured with the Geriatric Depression Scale (GDS), a 15-item questionnaire designed for use in the elderly population [52]. The instrument contains 15 items that are answered “yes” or “no”, and a score of 0–4 is considered “normal”, 5–10 is “suggestive of mild depression”, and 11–15 is “suggestive of severe depression” [52]. Considered a reliable instrument, Cronbach’s α is 0.71 in the elderly population [51]. Cronbach’s α was 0.79 in this study.

Loneliness was measured with the 20-item revised University of California at Los Angeles (UCLA) Loneliness Scale designed to measure general feelings of social isolation and dissatisfaction with one’s social interactions [53]. The 20-item, Likert style questionnaire contains 10 positively worded items and 10 negatively worded items. After reversing the negatively worded items, all items are summed and a total score is obtained ranging from 20 to 80. Higher scores indicate higher levels of loneliness. Considered a reliable instrument, Cronbach’s α is 0.86 in the elderly population [51]. Cronbach’s α was 0.91 in this study.

Religious coping was measured with the Brief RCOPE. The 14-item questionnaire is designed to measure the role religious practices serve to cope with life stressors. The instrument consists of seven items that indicate positive religious coping patterns, and seven items that indicate negative religious coping patterns. The scores on each subscale are summed, reported separately, and range from 7 to 28. The Brief RCOPE is considered reliable when used in adults from various cultures and religious affiliations, and Cronbach’s α is 0.81 [54]. Cronbach’s α was 0.72 in this study.

For biomarker assessment, each participant provided 1–2 mL saliva sample between 1:00 P.M. and 5:00 P.M. to control for circadian rhythmicity, and was collected after the standardized questionnaires were completed. Most participants successfully provided saliva within 10–30 min. Saliva samples were secured in a collection tube, placed in a biohazard laboratory bag inside a secure cooler with ice, and delivered to the Biosciences Laboratory at the University. Saliva samples were stored in −80 degree Celsius until analyzed for cortisol and IL-1β.

To evaluate precision of biological measures, coefficients of variability (CV) were calculated. The intra-assay CV was calculated from CVs of different plates per manufacturer instructions. The intra-assay coefficient of variation (CV) was calculated from the duplicates and inter-assay CV was calculated from controls on different plates. *A-priori* criterion for intra-assay CV was <10% and inter-assay CV was <15%, which were met for all salivary biomarkers [55]. Saliva samples were assayed using standardized enzyme immunoassay (EIA) kits [55]. For cortisol, intra-assay CV is 4%–7% and inter-assay CV is 3%–11%. Sensitivity of the EIA kit is 0.0007 µg/mL. For IL-1β, intra-assay CV is 2%–3% and inter-assay CV is 4.5%. Sensitivity of the EIA kit is 0.6 pg/mL.

### 2.4. Data Analysis

Descriptive statistics were completed for all study variables including demographic information. As spiritual pain was measured on an ordinal scale, bivariate relationships with psychometric and biologic measures were evaluated with the Spearman correlation coefficient. A priori *p* value was set at ≤0.05. Data were analyzed using SAS 9.4 for Windows.

## 3. Results

### 3.1. Participant Characteristics

The sample was comprised of 88 MOW clients (Table 1). Age ranged from 60 to 95 years (*M* = 75.4, *SD* = 9.0) and the majority were high school educated, Caucasian females who reported a variety of medical problems. The most commonly reported medical problems were hypertension, hypercholesterolemia, diabetes mellitus type 2, coronary artery disease, depression, and anxiety. Few participants reported thyroid problems, osteoarthritis, and migraines. Most participants reported using less than seven prescriptions per day and only a few reported daily vitamin or herbal remedy usage.

**Table 1:** Characteristics of the study population (*N* = 88).

	N (%)	Mean	SD	Range
**Age (years)**	88	75.4	9.0	60–95
Males		74.9	10.1	60–95
Females		75.6	8.5	60–94
**Gender**				
Males	30 (34.0)			
Females	58 (66.0)			
**Race**				
Caucasian	83 (94.3)			
Black	5 (5.7)			
Other	0			
**BMI**				
Males	30 (34.0)	28.5	6.54	17.9–46.9
Females	58 (66.0)	30.6	7.7	13.7–51.5
**Marital Status**				
Married	26 (29.5)			
Widowed	29 (33.0)			
Divorced	25 (28.4)			
Single	8 (9.1)			
**Education (years)**	88	12.39	2.25	7–18

### 3.2. Levels of Psychosocial Factors and Biological Responses

As shown in Table 2, overall mean scores for spiritual pain were low (*M* = 1.56, *SD* = 0.89). On the 4-point Likert scale for spiritual pain, *n* = 58 (65%) participants rated spiritual pain as 1 (none), *n* = 17 (19%) rated it as 2 (somewhat), *n* = 8 (9%) rated it as 3 (quite a bit), and *n* = 5 (6%) rated spiritual pain as 4 (a great deal). Overall mean scores for the PSS indicated low level of stress (*M* = 15.02, *SD* = 6.21), while mean scores for the GDS (*M* = 4.31, *SD* = 3.02) and R-UCLA (*M* = 39.9, *SD* = 12.22) indicated minimal depression and moderate loneliness. Mean scores for positive religious coping were high (*M* = 21.98, *SD* = 5.04), whereas mean scores for negative religious coping were low (*M* = 9.72, *SD* = 3.35). Overall levels of cortisol were within the expected range for adults (*M* = 0.33 µg/dL, *SD* = 0.79), however, overall levels of IL-1β were higher than the expected range (*M* = 561.73 pg/mL, *SD* = 830.42).

**Table 2:** Descriptive data for psychometric and biologic measures (*N* = 88).

	Possible Range	Score Range	Mean	SD
**Spiritual Pain**	1–4	1–4	1.56	0.89
Males		1–4	1.57	0.86
Females		1–4	1.55	0.91
**Stress**	0–40	0–38	15.02	6.21
Males		3–26	13.40	5.55
Females		0–38	16.64	13.33
**Depression**	0–15	0–13	4.31	3.02
Males		0–11	3.63	3.03
Females		0–13	4.99	3.32
**Loneliness**	20–80	20–67	39.9	12.22
Males		20–63	39.40	11.20
Females		20–67	40.47	13.33
**+Relig. Coping**	7–28	7–28	21.98	5.04
Males		11–28	21.03	5.00
Females		7–28	22.93	5.76
**−Relig. Coping**	7–28	7–25	9.72	3.35
Males		7–18	9.49	3.19
Females		7–25	9.95	3.79
**Cortisol µg/dL**				
**Both Genders**		0.01–5.17	0.33	0.76
Males		0.03–5.17	0.58	1.30
Females		0.01–1.75	0.19	0.24
**IL-1β pg/mL**				
**Both Genders**		6.0–5253.19	554.86	872.44
Males		6.0–5253.19	638.16	1142.06
Females		6.0–3592.11	485.19	607.82

*Note*: +Relig. Coping = positive religious coping patterns; −Relig. Coping = negative religious coping patterns.

### 3.3. Correlations of Spiritual Pain

Correlations of spiritual pain with psychosocial and biological data are presented in Table 3. Spiritual pain was positively and significantly correlated with stress (*r* = 0.35), depression (*r* = 0.27), and negative religious coping (*r* = 27; all *p* ≤ 0.01). Correlations of spiritual pain with loneliness, positive religious coping, cortisol, and IL-1β were non-significant.

**Table 3:** Correlation coefficient for psychometric and biological data (*N* = 88).

	Spiritual Pain	*p* Value
Stress	*r* = 0.35	≤ 0.001
Depression	*r* = 0.27	0.01
Loneliness	*r* = 0.15	0.16
+Religious Coping	*r* = −0.12	0.27
−Religious Coping	*r* = 0.27	0.01
Cortisol	*r* = 0.02	0.87
IL-1β	*r* = −0.07	0.50

## 4. Discussion

The primary objective of this cross-sectional study was to examine spiritual pain from a biobehavioral perspective to better understand associations with psychosocial factors (stress, depression, loneliness, and religious coping) and salivary biomarkers of stress and inflammation (cortisol and IL-1β) in MOW clients.

### 4.1. Stress, Depression, and Religious Coping

The hypothesis that spiritual pain would correlate positively with stress, depression, and negative religious coping was supported in this study. Our significant and positive correlations of spiritual pain with stress and depression are similar with findings in other studies. In Delgado-Guay’s study (2011) with advanced cancer patients (*N* = 100) in an acute care setting, correlations approached statistical significance to show for greater spiritual pain, greater anxiety, and greater depression [11]. Mako *et al*. (2006) reported a significant and positive correlation of spiritual pain and depression, but not physical pain or severity of illness in a sample of patients with advanced cancer in a palliative care hospital [12]. In addition to their quantitative analysis, Mako *et al*. (2006) asked participants to qualitatively describe their spiritual pain and responses were placed into three categories [12]. First, 48% of participants framed their spiritual pain in intra-psychic terms, such as suffering with despair, loss, regret, and anxiety. The next category was related to the divine, whereas 13% of participants described feeling abandoned by God, being without faith, and/or a religious or spiritual community. Lastly, 38% of participants described spiritual pain as it related to interpersonal feelings, such as feeling unwanted by family members or feeling disconnected from others. These qualitative descriptions of spiritual pain, coupled with mixed findings in the literature, indicate the complexity of the concept and need for additional research to better understand spiritual pain as it relates to health.

Our findings are not consistent with published evidence on spiritual pain and religious coping. Delgado-Guay *et al*. (2011) did not find a significant correlation between spiritual pain and religious coping [11]. Harrison *et al*. (2001), however, reported that the use of positive religious coping strategies was related to higher self-esteem, life satisfaction, and quality of life, whereas less use of religious coping strategies was related to more stress and depressive symptoms [56]. In a meta-analysis, religious coping was negatively correlated with negative psychosocial symptoms, such as stress and depression [37]. Musick and Wilson (2003) examined participation in religious activities and depression in a large, population-based study of community dwelling adults over an 8-year period and reported that higher involvement in religious activities was associated with less depression over time, but only in those ≥65 years old [57]. Authors who conducted another large, population-based study in community dwelling adults reported religion was significantly and negatively correlated with depression [58]. However, other studies report significant and positive associations of religious coping and negative psychosocial symptoms [59,60] or non-significant associations of religious coping and negative psychosocial symptoms [61,62]. Because MOW clients are at high risk for feelings of stress and depression, it is not surprising that negative religious coping in this population would correlate positively with spiritual pain.

### 4.2. Loneliness

The hypothesis that spiritual pain would correlate positively with loneliness was not supported. Although studies exist that examine spirituality and loneliness, we were unable to locate any published studies that specifically examined spiritual pain and loneliness. Koenig *et al*. (2012), however, propose a theoretical model to explain relationships of mental health and religion [2]. Interestingly, the model proposed by Koenig *et al*. (2012) is similar to the biobehavioral interaction model (Kang *et al*., 2010) used as a theoretical basis for this study [2,45]. The models differ, however, in that Koenig *et al*.’s (2012) model is specific for religion, spirituality, and health, whereas Kang *et al*.’s (2010) biobehavioral interaction model is general and can be adapted for use with almost any combination of variables [2,45]. In Koenig *et al*.’s (2012) model, negative aspects of mental health are characterized by the absence of positive emotions and positive cognitive states. Thus, positive mental health is strongly and inversely related to negative mental states [2]. For example, high religious and spiritual beliefs are associated with positive mental states, such as peace, harmony, well-being, happiness, and joy. On the other hand, low religious and spiritual beliefs are associated with negative mental states, such as depression, anxiety, suicide, psychosis, and personality disorders [2]. According to the model, religion and spirituality are expected to correlate negatively with loneliness, whereas spiritual pain would expect to correlate positively. In a systematic review, however, 17 studies on religion, spirituality, and loneliness were analyzed and findings were not consistent. Five authors reported significant and negative correlations of religion, spirituality, and loneliness, five others reported mixed or non-significant findings, and two authors reported significant and positive correlations [2]. Given inconsistent published findings on correlations of religion, spirituality, and loneliness, as well as our non-significant findings of spiritual pain and loneliness, additional research is needed to better understand influences of these variables on loneliness.

### 4.3. Cortisol and IL-1β

Similar to loneliness, we were not able to locate published studies on correlations of spiritual pain with biomarkers of stress and inflammation. Some researchers, however, have attempted to examine the relationship of religion, spiritualty, and biomarkers of the immune system. In a comprehensive review of the literature, Koenig (2011) reported that 56% of 25 studies indicated positive correlations of religion, spirituality, and immune function [63]. Koenig (2011) also located 29 studies that examined religion, spirituality, and cortisol and found that 19 (66%) of those studies reported negative correlations of religion, spirituality, and cortisol levels [63]. Given the lack of published findings on correlations of spiritual pain and biomarkers of immune function, as well as our non-significant findings with spiritual pain, additional research is needed to better understand influences of these variables on spiritual pain.

## 5. Clinical Implications

The experience of spiritual pain is complex, and may be a part of the older adult’s life experience due to loss of independence, chronic illness, or perceived life crisis. For these reasons, spiritual pain must be identified and treated [6]. A comprehensive spiritual assessment should be performed and include a screening of spiritual pain, and findings from the assessment should be included in the overall management of health. Identification of the presence of spiritual pain will provide clinicians with the opportunity to incorporate resources such as prayer, relaxation techniques, spiritual rituals, and other complementary modalities of the patient’s religious tradition in the treatment plan [6]. A comprehensive spiritual assessment promotes a holistic approach to caring for the whole person, and is important for the interdisciplinary team as it encourages the person’s involvement in the self-management [6].

The person who performs the spirituality assessment is not required to be the person who meets the spiritual needs, but is the person who identifies spiritual needs and arranges for specific spiritual resources or interventions [6]. In many acute or hospital settings, the chaplain is the expert in spiritual care and is the person who will address spiritual needs. It is ideal for the chaplain to perform an ongoing evaluation of the spiritual treatment plan and keep the interdisciplinary team apprised of outcomes. Ongoing evaluation of spiritual pain includes monitoring the person’s coping, peacefulness, cooperativeness, and verbalization of inner conflicts, reconciliation needs, grief, losses, or regrets [6]. In a nursing home or home setting, it may be a friend, family member, or nurse who addresses spiritual needs [6]. A comprehensive spiritual assessment is particularly important in the nursing home or home setting where a chaplain is not always easily accessed and spiritual needs may go unaddressed [2].

## 6. Conclusions

Although religious and spiritual practices are commonly used in the elderly, their influence on health is not well understood [37]. Our findings indicate significant and positive correlations of spiritual pain with stress, depression, and religious coping, but non-significant correlations of spiritual pain with loneliness, salivary cortisol, and salivary IL-1β. These findings are similar to the generally mixed findings in the literature of this field, indicating the complex nature of spiritual pain and its association with psychosocial and biological variables. Despite mixed findings, clinicians should perform a comprehensive spiritual assessment and include findings in the overall treatment plan for the older adult. The findings of this study may serve to fill gaps in the literature, particularly in relation to biobehavioral interactions surrounding spiritual pain and spirituality. Future research should be expanded to other biobehavioral variables in larger samples of diverse and vulnerable populations. Collective findings may be able to identify particularly vulnerable subgroups of population ultimately with more tailored interventions.

## 7. Limitations

This study has several limitations. The cross-sectional research design limits causal data analysis and interpretation of findings. The correlational associations between variables are not necessarily an indication of causality. The sample size was small, and the population was homogenous with a majority being Caucasian females. The sample did not undergo a clinical assessment for dementia, but instead we relied on self-report of neurodegenerative disease. In addition, the findings are not generalizable because we do not have access to demographic information for the entire population of MOW clients in this county. For these reasons, the findings of this study should be interpreted with caution. Despite these limitations, we present important insights on correlates of spiritual pain, stress, depression, religious coping, and salivary biomarkers of stress and inflammation in a disadvantaged elderly population of MOW clients. The findings of this study may significantly contribute to exploration of biobehavioral interactions in spirituality and health research.
